# Investigating Factors Associated with Depression of Type 2 Diabetic Retinopathy Patients in China

**DOI:** 10.1371/journal.pone.0132616

**Published:** 2015-07-07

**Authors:** Xujuan Xu, Xiaoyan Zhao, Duo Qian, Qing Dong, Zhifeng Gu

**Affiliations:** 1 Department of Nursing, Affiliated Hospital of Nantong University, Nantong, China; 2 School of Nursing, Nantong University, Nantong, China; 3 Department of Rheumatology, Affiliated Hospital of Nantong University, Nantong,China; Peking University, CHINA

## Abstract

**Aims and objectives:**

To assess the depression status of type 2 diabetic retinopathy patients in Nantong China and to identify factors associated with depression.

**Methods:**

Two hundred and ninety-four patients with type 2 diabetic retinopathy were recruited from the Affiliated Hospital of Nantong University. The severity of DR was measured in the worse eye. Depressive symptoms were assessed with the Center for Epidemiologic Studies Depression Scale (CES-D); the quality of life was measured with the Medical Outcomes Study Short Form 36 (SF-36). The logistic regression analyses were used to identify the independent factors of depression.

**Results:**

The mean age of the study subjects was 57.77 years (SD: 9.64). Approximately 35.7% of subjects reported depressive symptoms (n = 105).Multiple logistic regression analyses showed that female gender (p = 0.014), low monthly income (p = 0.01), poor vision in the better eye (P = 0.002), laser treatment history (p = 0.01) were significant risk factors for depression. The quality of life of individuals with CES-D score<16 was significantly better compared with individuals with CES-D score≥16.

**Conclusion:**

The reported depressive symptoms among type 2 diabetic retinopathy population is higher in Nantong China. Gender, salary, vision acuity and treatment history were important risk factors linked to this disorder in the Chinese type 2 diabetic retinopathy population from Nantong. More attention by medical care personnel needs to be paid to the psychological health of this population.

## Introduction

As the leading cause of blindness among working aged adults [1], Diabetic retinopathy (DR) is a serious threat to the quality of life for millions worldwide. A published analysis of data estimated that the overall prevalence was 34.6% for any DR[2]. In China, the prevalence of DR in subjects with high risk for diabetes was 14.9% and in the pre-diabetic population was 2.5%[3, 4]. Longer diabetes duration and poorer blood glucose management are strongly associated with DR[2]. However, performance of blood glucose self-management behaviors by patients with diabetes is suboptimal[5]. The prevalence of diabetes is rising notably in China[6, 7], and the rate of DR will increase rapidly.

Depression is a global public health problem, and its prevalence is increasing every year. Current epidemiological evidence suggests that people with diabetes have an increased risk of experiencing a depressive condition in their lifetime. A meta- analysis indicated that the presence of diabetes doubles the odds of comorbid depression[8]. De Groot et al[9] found depression was significantly associated with a variety of diabetes complications. Moreover, Mathew et al. found that visual impairment has been linked with depressive symptoms[10]. Individuals with both diabetes and visual impairment are likely to experience a considerable number of depressive symptoms.

Depression can lead to worse diabetes control, lower adherence to treatment, and increased economic burden of health care costs. One study indicated that persons with depressive symptoms were 3 times more likely to exhibit decreased adherence[11]. Another study has shown that depressed patients have greater levels of fatigue and anxiety, poorer quality of life, and more suicidal ideation[12]. All of these highlight the fact that depression must be understood in order to discover ways to prevent and treat it.

Existing studies have documented that the incidence of depression in diabetes was associated with gender, socioeconomic status, blood glucose level and insulin therapy[13–16]. A cross-sectional study conducted in urban Nepal showed 1-unit (%)increase in HbA1c was associated with a 2-point increase in depression score. Camara A et al.[15] suggests that male whose HbA1c≥9.0% had higher depression rate. Therefore we were specifically interested in the relationship between those variables and depression in DR patients. However, factors associated with depressive symptoms among type 2 diabetic retinopathy patients in Nantong have not yet been fully explored.

The aim of this study was to identify risk factors for depression among persons with type 2 diabetic retinopathy and to identify warning signs for early diagnosis and treatment for mood symptoms in these patients. It was hypothesized that some demographic (like gender, socioeconomic status) or disease-specific variables would affect the depressive symptoms.

## Materials and Methods

This was an observational cross-sectional study of consecutive patients with type 2 diabetic retinopathy from the Affiliated Hospital of Nantong University. Two hundred and ninety-four patients with type 2 diabetic retinopathy were recruited from November 2013 to June 2014. All participants fulfilled the 1999 World Health Organization (WHO) criteria for the diagnostic of type 2 diabetes[17]. The diagnosis of diabetic retinopathy was confirmed by mydriatic fundus examination and fluorescence angiography. The exclusion criteria for patients were: age <18 or >80 years at the time of interview; hearing and cognitive impairment; with co-morbidities and other complications (e.g., cancer, renal failure, glaucoma etc) that could seriously influence patients’ quality of life; any psychotropic drugs use during the past month; and not completing the questionnaire. Using the worse eye, we categorized the severity using the American Academy of Ophthalmology (AAO) clinical severity scale into mild non proliferative diabetic retinopathy(NPDR); moderate NPDR; severe NPDR; and proliferative diabetic retinopathy(PDR)[18]. Visual acuity was assessed using a 5-m standard logarithmic chart. Patients were asked to use their usual spectacles or a pinhole to obtain the best visual acuity at the retina. This 5-m standard logarithmic chart score ranging from 0.1 to 2.0, with higher scores indicating better visual acuity. If patients fail to see 0.1, they were asked to move forward, until could see 0.1,and visual acuity = 0.1* distance/5 (i.e., 4m:0.08; 3m: 0.06).Other variables included age, sex, monthly income, employment status, marital status, education, insurance, insulin use, hypertension, treatment history, depressive symptoms, duration of diabetes, and fasting plasma glucose. All participants consented to participate in this study. The study was approved by the ethical committees of the Affiliated Hospital of Nantong University(2013–045). After signing an informed consent form, subjects completed the study questionnaire either independently or with the help of the investigator in a face-to-face interview.

Center for Epidemiologic Studies Depression Scale(CES-D) was a reliability and validity scale, and it was used to screen for depressive symptoms[19]. Chinese version CES-D had been demonstrated to have high internal consistency (Cronbach's α = 0.90) and construct validity (CFI = 0.90;GFI = 0.948)[20]. Chinese version self-report scale had 20 questions, and indicated the presence and severity of depressive symptoms over the previous week. Each question had four-point response (ranging from rarely or none of the time to most or all of the time), with total scores ranging from 0 to 60. The scale scores greater than 16 suggested minor depression. Although the minor depression do not indicated a diagnosis of clinical depression, it has previously been shown to effectively identify individuals with clinically significant levels of depression in other studies[21].A study comparing several depression screening tools, indicated that the CES-D had the best ability to discriminate between depression and other non-depressive symptoms[22].

Participants’ health status was assessed using the Medical Outcomes Study Short Form 36 (SF-36)[23]. The SF-36 comprised eight multi-item dimensions: physical functioning (PF); role limitations due to physical problems (RP); role limitations due to emotional problems (RE); social functioning (SF); mental health (MH); energy/ vitality(VT); body pain (BP); and general health perception (GH). These eight multi-item dimensions are divided into two summary scores: the physicalcomponent summary (PCS) and the mental component summary (MCS).Scores were expressed on a 0–100 scale, with higher values indicating better quality of life. The questionnaire was culturally adapted and translated into Chinese. “Convergent validity and discriminant validity were satisfactory for all except the social functioning scale. Cronbach’s α coefficients ranged from 0.72 to 0.88 except 0.39 for the social functioning scale and 0.66 for the vitality scale. Two weeks test-retest reliability coefficients ranged from 0.66 to 0.94” [24].

Data were analyzed using the SPSS17.0. Descriptive statistics were used to analyze background variables. Means±Sds for normally distributed variables, median and interquartile range (IQR)for nonnormally distributed. Categorical variables as frequencies (%).To examine which variables result in the depressive symptoms, bivariate analyses including the chi-square test and Mann-Whitney U-tests were used to examine the association between factors and depressive symptoms. Variables shown to be significant in the bivariate analysis were included in the logistic regression analysis to identify the independent factors of depression with odds ratios (ORs), and the corresponding 95% CIs. Statistical significance was set at P<0.05.

## Results

The predisposing characteristics of DR patients are shown in Table 1.A total of 294 subjects(146 men; 49.66%) completed the entire study. The mean age of the study subjects was 57.77 years(SD: 9.64). Almost 35.7% (n = 105) individuals in the sample group reported depressive symptoms. The CES-D score ≥16 were higher in women (45.27%) compared with men (26.03%)(χ^2^ = 11.85; p = 0.001). More than fifty percent of the patients who reported depressive symptoms were found with monthly income >3000(χ^2^ = 26.45; p = 0.000). In addition, the higher reported depressive symptoms was also found when the insurance was covered by rural cooperative medicare system (p = 0.048).

**Table 1:** Comparison of demographic factor of DR patients by depression status.

		Depression Status			
Variables	Overall sample	CES-D score ≥ 16	CES-D score < 16	χ^2^/t	P
Total, n(%)	294	105(35.70)	189(64.30)		
Gender, n(%)				11.85*	**0.001**
Male	146(49.66)	38(26.03)	108(73.97)		
Female	148(50.34)	67(45.27)	81(54.73)		
Age (year), mean (SD)	57.77(9.64)	56.77(10.56)	58.33(9.08)	1.33*	0.190
Employment status, n(%)				2.86	0.091
Working	137(46.60)	42(30.66)	95(69.34)		
Not working	157(53.40)	63(40.13)	94(59.87)		
Education, n(%)				1.49	0.480
Primary or below(0–6 years)	120(40.82)	46(38.33)	74(61.67)		
Secondary (7–13 years)	157(53.40)	55(35.03)	102(64.67)		
Tertiary (>13 years)	17(5.78)	4(23.53)	13(76.47)		
Marital status(Married),n(%)	282(95.90)	98(34.75)	184(65.25)	2.65	0.170
Monthly income(RMB),n(%)				26.45	**<0.001**
<1000	119(40.48)	62(52.10)	57(47.90)		
1000–2000	81(27.55)	22(27.16)	59(72.84)		
2000–3000	55(18.71)	16(29.09)	39(70.91)		
≥3000	39(13.27)	5(12.82)	34(87.18)		
Insurance, n(%)				6.06	**0.048**
No	26(8.84)	7(26.92)	19(70.08)		
Medical insurance	117(39.80)	34(29.06)	83(70.94)		
Rural Medicare cooperative	151(51.36)	64(42.38)	87(57.62)		

Bold values indicate significant results(p<0.05).

*Independent t-tests.

DR: Diabetic retinopathy

In Table 2, for CES-D score ≥16 patients, significant associations were found with vision in the better eye(p = 0.000), vision in the worse eye(p = 0.005) and treatment history (p = 0.021). However, no statistically significant associations were found with regard to the duration of diabetes(p = 0.71), fasting plasma glucose(p = 0.99), insulin use(p = 0.67) and severity of DR(p = 0.11).

**Table 2:** Comparison of clinical factor of DR patients by depression status.

		Depression Status			
Variables	Overall sample	CES-D score ≥ 16	CES-D score < 16	z/χ^2^	P
Duration of diabetes(year), median (IQR)	10.00(10.00)	11.00(12.00)	10.00(10.00)	-0.37	0.710
Fasting plasma glucose, mg/dl, median (IQR)	7.00(2.00)	7.10(2.20)	7.00(2.00)	-0.02	0.990
Postprandial glucose (2h),mg/dl, median (IQR)	9.00(3.20)	9.00(5.30)	9.10(2.95)	-1.18	0.240
HbA1c,%, median (IQR)	8.90(1.95)	8.35(3.05)	9.00(1.80)	-0.55	0.580
Vision in the better eye, median (IQR)	0.30(0.35)	0.25(0.30)	0.30(0.40)	-3.63	**0.000**
Vision in the worse eye, median (IQR)	0.08(0.15)	0.01(0.15)	0.10(0.20)	-2.84	**0.005**
Insulin use (yes),n(%)	197(67.01)	72(36.55)	125(63.45)	0.18*	0.670
Hypertension(yes), n(%)	169(57.48)	55(32.54)	114(67.46)	1.74*	0.190
Severity of DR, n(%)				5.95*	0.110
Mild NPDR	15(5.10)	3(20.00)	12(80.00)		
Moderate NPDR	31(10.54)	7(22.58)	24(77.42)		
Severe NPDR	27(9.18)	13(48.15)	14(51.85)		
PDR	221(75.17)	82(37.10)	139(62.90)		
Treatment history, n(%)				9.69*	**0.021**
No	181(61.56)	62(34.25)	119(65.75)		
Laser treatment	58(19.73)	29(50.00)	29(50.00)		
Operative treatment	34(11.56)	11(32.35)	23(67.65)		
Laser +Operative	21(7.14)	3(14.29)	18(85.71)		

Bold values indicate significant results(p<0.05).

IQR: Interquartile range

DR: Diabetic retinopathy

NPDR: Non proliferative diabetic retinopathy

PDR: Proliferative diabetic retinopathy

*Chi-square test.

Multiple logistic regression analyses were used to identify a model to predict DR patient who would have depression. The results showed that variables like gender, monthly income, vision in the better eye, and treatment history were significant risk factors for depression (Table 3). This model had a good fit under the Hosmer- Lemeshow goodness-of-fit test (R^2^ = 0.246, χ^2^ = 3.473,p = 0.901),and accuracy of the model was 71.4%.Women were 2.10 times more likely to be depressed than men (p = 0.014). Patients whose monthly income <1000 were 4.62 times more likely to be depressed than those whose monthly income were 3000 or above (p = 0.01). A one unit decrease in vision in the better eye was associated with the increase of depression rate(OR = 0.13,P = 0.002). Those who were reported with laser treatment history were 6.22 times more likely to be depressed than those who were reported with laser and operative treatment history (p = 0.01) on this factor.

**Table 3:** Logistic regression model of DR patients on depression.

					Exp(B)95%CI		
Variables	B	SE	Wald	OR	Lower bound	Upper bound	P value
Gender							
Female	0.74	0.30	6.01	2.10	1.16	3.80	**0.014**
Male	———	———	———	REF	———	———	**———**
Monthly income			11.35				**0.010**
<1000	1.53	0.59	6.83	4.62	1.47	14.57	**0.009**
1000–2000	0.50	0.58	0.75	1.65	0.53	5.16	0.390
2000–3000	0.96	0.60	2.58	2.61	0.81	8.38	0.110
≥3000	———	———-	———	REF	———	——-	———
Vision in the better eye	-2.01	0.66	9.43	0.13	0.04	0.48	**0.002**
Treatment history			8.98				**0.029**
No	1.17	0.67	3.06	3.22	0.87	11.93	0.080
Laser treatment	1.83	0.71	6.62	6.22	1.55	25.06	**0.010**
Operative treatment	0.78	0.76	1.04	2.18	0.49	9.70	0.310
Laser +Operative	———	———-	———	REF	———	———	——-

DR: Diabetic retinopathy

REF, reference group

Bold values indicate significant results(p<0.05).

The subjects were divided into two groups according to the CES-D score. An analysis showed that all the dimension scores of SF-36 in individuals with CES-D score <16 were significantly higher compared with individuals with CES-D score ≥ 16(p<0.001). The results are shown in Table 4.

**Table 4:** Comparison between the CES-D score ≥ 16 group and CES-D score < 16 group in SF-36 domain score.

QOL assessments (SF-36 domains),median (IQR)		Depression Status			
	Overall sample	CES-D score ≥ 16	CES-D score < 16	Z	P
PF(Physical functioning)	95.00(5.00)	90.00(10.00)	95.00(5.00)	-4.32	**0.000**
RP(Role-physical)	25.00(75.00)	00.00(25.00)	50.00(75.00)	-6.64	**0.000**
RE(Role-emotional)	100.00(33.33)	66.67(33.33)	100.00(0.00)	-6.71	**0.000**
BP(Bodily pain)	100.00(16.00)	84.00(26.00)	100.00(16.00)	-4.39	**0.000**
MH(Mental health)	56.00(24.00)	44.00(12.00)	64.00(21.00)	-11.02	**0.000**
VT(Vitality)	60.00(30.00)	40.00(30.00)	65.00(25.00)	-7.32	**0.000**
SF(Social functioning)	87.50(25.00)	75.00(37.50)	87.50(15.62)	-7.85	**0.000**
GH(General health)	40.00(10.00)	35.00(20.00)	40.00(10.00)	-6.57	**0.000**
PCS	250.00(96.00)	214.00(60.00)	282.00(80.25)	-7.66	**0.000**
MCS	297.50(94.50)	226.83(90.33)	315.50(53.63)	-9.65	**0.000**

Bold values indicate significant results(p<0.05).

IQR: Interquartile range

PCS: the physical component summary

MCS: the mental component summary

## Discussion

We found the risk of depression increases with the decreasing of household income and visual acuity. Importantly, we also found that the depressive symptoms were negatively associated with patients’ quality of life.

This study found that almost 35.7% of subjects had depressive symptoms (CES-D score ≥16), depression is becoming a serious problem. It is in line with other report[25]. DR patients were reported to feel frustrated, concerned about further vision loss and dissatisfied with life due to the restrictions on driving, transportation, social life, work and independence[26]. Devenney et al. [27] reported that even mild DR patients were found with uncertainty and vulnerability in the aspect of vision loss.

In the final multivariate logistic regression, gender and one’s mouth income had significant influence on CES-D scores. It was suggested that a lower income female supports a higher risk of depression among DR patients. Many epidemiological studies have also shown that women are at greater risk for major depression[28, 29]. Zhou X et al[30] found the prevalence of depression in Chinese women was higher than men. This increased prevalence may be because women may face more stress due to balancing work and home life demands, managing personal relationships while simultaneously attempting to manage their disease. Zhou X[30]suggested that household income is the main risk factor for depressive symptoms. In the United States, the cost of diabetic retinopathy was estimated to be more than $500 million per year [31, 32]. The increased disease burden can lead great psychological pressure among low-income groups. Moreover, combining with depression can also lead to the increased economic burden of health care costs. This adverse consequence will give rise to further psychosocial stress.

Additionally, the multivariate logistic regression analysis also indicated that vision in the better eye and treatment history were significantly associated with depressive symptoms. DR patients with visual impairment may face challenges in completing reading, working at home or in the office, walking, driving and interacting with others, which in some cases lead to mental health impairment[33,34]. Trento M et al.[35] confirm that patients with visual impairment due to DR experience discomforts in everyday life and lose autonomy in day-to-day functioning, with the loss of ability to perform specific tasks. Lamoureux EL et al. [36] reported that DR patients’ emotional reaction was affected of vision loss. A longitudinal analysis investigated that change in visual acuity was the important factor associated with change in mental health during a 10-year period[34]. Previous laser treatment was associated with further worsening in some of the indicators, especially those related to general health, vision and everyday activities, such as driving[35]. Bailey CC et al.[37] indicated that nine months after laser photocoagulation, patients thought that the ability to do things was worse than before treatment. Possibly, this after-effects of laser treatment is the reason for our results.

There are several limitations in our research. First, our sample was selected from one hospital in China for convenience, so generalization of the findings to other population should be cautious. Second, we used self-reported and closed-ended questionnaires. Third, the sample sizes of patients with NPDR were small.

In conclusion, the present study provides that depressive symptoms are common in DR patients. The monitoring of depression should be embedded in health care. Gender, salary, vision acuity and treatment history play an important role in the incidence of mental health, and these changes might be a potential avenue for developmentally and culturally appropriate interventions to type 2 diabetic retinopathy combined with depression. Education about the connection between physical health and mental health should be considered for psycho-education programs within female gender, low monthly income, and poor vision population. Moreover, the government should raising the allowance and providing more employment opportunity for low-income earners to improve their quality of life.
